# QuantiFERON-TB Gold Plus CD8+ T cell responses in contacts with tuberculosis disease and recent tuberculosis infection

**DOI:** 10.1128/spectrum.01353-25

**Published:** 2025-10-20

**Authors:** Cynthia Bin-Eng Chee, KhinMar Kyi-Win, Shera Tan, Yee-Tang Wang

**Affiliations:** 1 Department of Respiratory and Critical Care Medicine, Tan Tock Seng Hospital 63703, Singapore, Singapore; 2 National Centre for Infectious Diseases 534955 , Singapore, Singapore; Geisel School of Medicine at Dartmouth, Lebanon, New Hampshire, USA

**Keywords:** QuantiFERON Plus, CD8 T cells, interferon gamma release assay, latent TB infection, tuberculosis

## Abstract

The QuantiFERON-TB Gold Plus (QFT-Plus) replaced the QuantiFERON-TB Gold in-tube (QFT-GIT) in 2016 to increase assay sensitivity, based on the role of *Mycobacterium tuberculosis*-specific CD8+ T cells in the host response to tuberculosis infection (TBI). The QFT-Plus incorporates a fourth tube (TB2 tube) in addition to the TB antigen tube of the QFT-GIT (TB1 tube). The TB2 tube contains shorter peptides from early secretary antigenic 6 kDa (ESAT-6) and culture filtrate protein 10 (CFP-10) to stimulate CD8+ T cells as well as long peptides contained in the TB1 tube. Evidence that *Mycobacterium tuberculosis*-specific CD8+ T cells preferentially recognize heavily infected cells has generated interest in the QFT-Plus CD8+ T cell response (taking TB2-TB1 interferon-gamma [IFN-γ] >0.6 IU/mL as a threshold for CD8+ response) as a marker for TB disease and for recent TBI. We retrospectively analyzed the CD8+ responses in the QFT-Plus results of contacts screened at the Singapore TB Contact Clinic from January 2018 to October 2018. We found significantly higher proportions of contacts with TB2-TB1 IFN-γ >0.6 IU/mL among those with TB disease (28.8% vs 12.6%, *P* < 0.0001) and Stringent Converters (23.3% vs 12.6%, *P* < 0.0001) compared to All Others with TBI. Median TB2-TB1 IFN-γ values were also significantly higher in those with TB disease (0.13 vs 0.06 IU/mL, *P* < 0.0001) and Stringent Converters (0.17 vs 0.06 IU/mL, *P* = 0.036) compared to All Others with TBI. Our findings add to the evidence that the CD8+ response measured by the QFT-Plus assay may predict TB disease and recent TB infection.

**IMPORTANCE:**

Evidence that *Mycobacterium tuberculosis*-specific CD8+ T cells preferentially recognize heavily infected cells has generated interest in the QuantiFERON-TB Gold Plus (QFT-Plus) CD8+ T cell response as a marker for tuberculosis (TB) disease and for recent TB infection. Taking TB2-TB1 interferon-gamma >0.6 IU/mL as a threshold for CD8+ response, we found CD8+ T cell response to be associated with TB disease and stringent QFT-Plus conversion in a cohort of contacts screened under our country’s national program. This adds to the evidence for the potential utility of this marker to identify persons who would benefit from further investigations for TB disease and those with recent infection who would be candidates for TB preventive treatment.

## INTRODUCTION

It is estimated that tuberculosis infection (TBI) affects 1.7 billion people, 23% of the world’s population (1). A pillar of the World Health Organization End TB Strategy is that of targeted TBI screening to identify persons eligible for TB preventive treatment (TPT) (2). Current TB screening tests, such as the tuberculin skin test (TST) and the more specific interferon-gamma release assays (IGRAs), which measure CD4+ T cell responses to early secretary antigenic 6 kDa (ESAT-6) and culture filtrate protein 10 (CFP-10), have thus far not been shown to discriminate between persons with TB disease and TBI, nor to identify persons with recently acquired TBI (an important risk factor for progression to TB disease) (3).

The QuantiFERON-TB Gold in-tube (QFT-GIT) (Qiagen, Hilden, Germany) was replaced with the QuantiFERON-TB Gold Plus (QFT-Plus) in 2016 to increase assay sensitivity, based on the role of *Mycobacterium tuberculosis (M. tb*)-specific CD8+ T cells in the host response to TB infection (4–6). The QFT-Plus incorporates a fourth tube (TB2 tube) in addition to the TB antigen tube of the QFT-GIT (TB1 tube), which contains long peptides from ESAT-6 and CFP-10 that stimulate CD4+ T cells (7). The TB2 tube contains shorter peptides from ESAT-6 and CFP-10 to stimulate CD8+ T cells as well as the long peptides contained in the TB1 tube. Although two systematic reviews and meta-analyses found minimal differences in sensitivity and specificity between the QFT-Plus and QFT-GIT (8, 9), there is interest in using the QFT-Plus CD8+ T cell response to identify persons with TB disease and recent TBI, in view of evidence that *Mycobacterium tuberculosis*-specific CD8+ T cells preferentially recognize heavily infected cells (3). Investigators evaluating the QFT-Plus CD8+ response have used a differential between the TB2 and TB1 tubes of interferon-gamma (IFN-γ) >0.6 IU/mL to indicate CD8+ T cell response based on the QFT-GIT assay’s limit of variability of 0.6 IU/mL (10).

Despite a presumed high background rate of TBI in our intermediate TB incidence country, Singapore implemented TPT for close contacts with TBI in 1998 as one of several TB control measures under the Singapore TB Elimination Programme (11–13). The TST was used to identify persons with TBI. This was replaced by the QFT-GIT in stages from 2006, and the QFT-Plus from December 2017. We retrospectively analyzed the CD8+ responses in the QFT-Plus results of contacts screened at the TB Contact Clinic (TBCC) from January 2018 to October 2018, focusing on its utility in predicting TB disease and recent TBI.

## MATERIALS AND METHODS

### Patients and recruitment

TB is a notifiable disease in Singapore. Identified close contacts of sputum acid-fast bacilli smear or *Mycobacterium tuberculosis* complex culture-positive pulmonary or laryngeal TB cases are screened for TB disease and TBI at the TBCC, which performs nationwide contact screening. During the study period, the QFT-Plus was performed for immunocompetent contacts ≥5 years old, the T-SPOT.*TB* was used for immunocompromised contacts, and the TST for contacts <5 years of age. For the QFT-Plus, blood was collected in four heparinized 1 mL tubes provided in the kit procured from the manufacturer. The assay was performed at the Tan Tock Seng Hospital Microbiology Laboratory and interpreted according to the manufacturer’s instructions.

Contacts who tested QFT-Plus negative within 8 weeks of last exposure to the index case (ie. the window period for test conversion) were retested 8 or more weeks after exposure. Chest radiography was performed for all IGRA-positive contacts and for those who were symptomatic or who had additional risk factors for TB disease (e.g., immunocompromised state, diabetes mellitus, being elderly) regardless of IGRA result.

The screened contacts were categorized into those with “TB disease,” defined as those with chest radiograph abnormalities compatible with pulmonary TB; “Stringent Converters,” i.e., those most likely to have recent TBI; and “All Others with TBI” (i.e., those who tested positive on their first QFT-Plus and non-stringent converters in the absence of active TB), many of whom may have remote infection. “Stringent Converters” were defined as contacts whose post-window QFT-Plus test demonstrated an increase from IFN-γ <0.35 IU/mL (a negative result) to IFN-γ >0.7 IU/mL in either or both the TB1 or TB2 tubes in the absence of TB disease. This was to more accurately identify contacts with true test conversion (and hence recent TBI) by excluding those whose second QFT-Plus results fell within 0.2 to 0.7 IU/mL, the so-called “zone of uncertainty” in which test readings may be due to within-subject or test variability (14). We compared the qualitative (positivity in the TB1 and/or TB2 tubes) and quantitative QFT-Plus responses, TB1-Nil (TB1), TB2-Nil (TB2), and the CD8+ responses as indicated by TB2-TB1, between contacts with TB disease and All Others with TBI, and between Stringent Converters and All Others with TBI.

### Statistical analysis

The χ^2^ test or Fisher’s exact test, as appropriate, was used to compare categorical variables, while the Mann-Whitney *U*-test was applied to compare continuous variables. Crude and adjusted odds ratios (ORs) with their corresponding 95% confidence intervals (CIs) were calculated using logistic regression analyses to identify factors associated with the CD8 response (TB2-TB1 IFN-γ >0.6 IU/mL).

All *P* values were two-sided, and statistical significance was defined as *P* < 0.05. Statistical analyses were conducted using IBM SPSS Statistics, version 26.0.

## RESULTS

**Fig 1:**
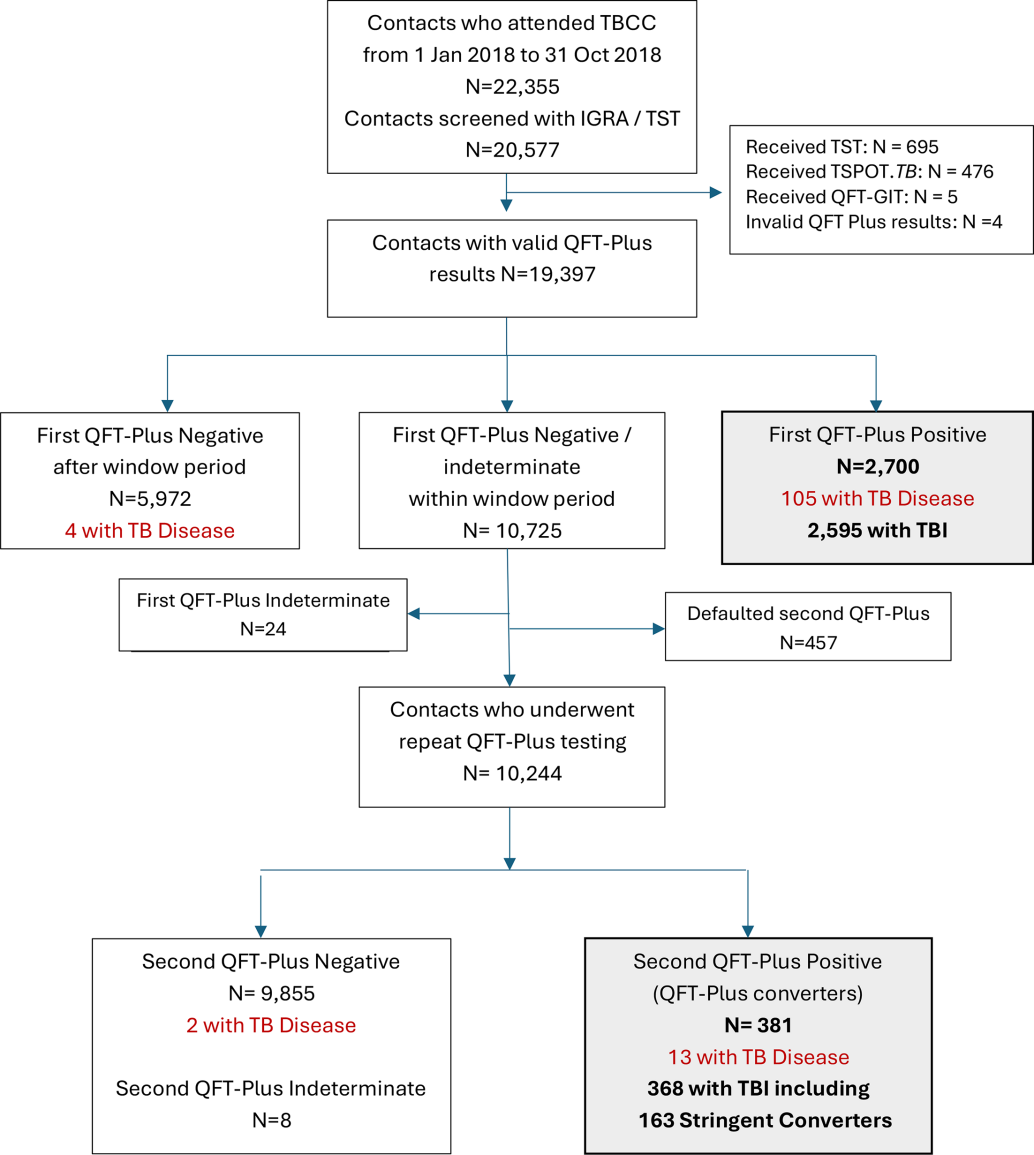
Flow chart of screening outcome of contacts at the TBCC from 1 January 2018 to 31 October 2018. Over the study period, 22,355 contacts attended the TBCC, of whom 20,577 underwent an IGRA or TST. Among the 19,397 contacts with valid QFT-Plus results, 2,700 (13.9%) tested positive on their first test, including 105 with TB disease. Of the 10,244 QFT-Plus-negative contacts who underwent post-window period testing, 381 (3.7%) tested positive on their second test, including 13 with TB disease. Six contacts who tested QFT-Plus negative had TB disease. Of the 368 QFT-Plus converters, 163 (44.3%) were Stringent Converters.

Over the study period, 22,355 contacts attended the TBCC, of whom 20,577 underwent an IGRA or TST. Among the 19,397 contacts with valid QFT-Plus results, 2,700 (13.9%) tested positive on their first test, including 105 with TB disease. Of the 10,244 QFT-Plus-negative contacts who underwent post-window period testing, 381 (3.7%) tested positive on their second test, including 13 with TB disease. Six contacts who tested QFT-Plus negative had TB disease. Excluding cases of TB disease, there were 2,595 and 368 contacts who tested QFT-Plus positive on their first and repeat tests, respectively, and who were therefore deemed to have TBI. Of the 368 test converters, 163 (44.3%) demonstrated an increase of IFN-γ to >0.7 IU/mL from a negative result in either or both the TB1 or TB2 tubes (“Stringent Converters”) (Fig. 1). The demographics of the contacts according to screening outcome (TB disease/TBI/no TBI) are shown in Table 1. The exposure settings for the contacts were household (22%), workplace (29%), congregate settings such as prisons and schools (32%), and others (17%).

**TABLE 1:** Demographics of contacts screened according to screening outcome

	TB disease (118 QFT-Plus positive) *N* = 124 (%)	TBI QFT-Plus positive *N* = 2,963 (%)	No TBI QFT negative/indeterminate *N* = 15,853 (%)
Gender			
Male	76 (61.3)	1,691 (57.1)	8,323 (52.5)
Female	48 (38.7)	1,272 (42.9)	7,530 (47.5)
Country of birth			
Foreign-born	54 (43.5)	1,361 (49.5)	5,844 (36.9)
Local-born	70 (56.5)	1,602 (54.1)	9,922 (62.6)
Unknown	0 (0)	0 (0)	87 (0.5)
Age group (years)			
Median (range)	43.5 (13.8-85.8)	45.2 (1.6-100.5)	34.9 (1.7-104.3)
<5	0 (0.0)	3 (0.1)	68 (0.4)
5–14	1 (0.8)	74 (2.5)	1,034 (6.5)
15–59	91 (73.4)	2,158 (72.8)	12,520 (79.0)
≥60	32 (25.8)	728 (24.6)	2,231 (14.1)
Smoking history			
Yes	32 (25.8)	445 (15.0)	2,148 (13.9)
No	91 (73.4)	2,457 (82.9)	13,338 (84.1)
Unknown	1 (0.8)	61 (2.1)	367 (2.3)
Diabetes mellitus			
Yes	9 (7.3)	283 (9.6)	913 (5.8)
No	115 (92.7%)	2,680 (90.4%)	14,940 (94.2%)

### Qualitative and quantitative QFT-Plus results

Among the 124 with TB disease, 118 tested QFT-Plus positive, giving a test sensitivity of 95.2%. None of the six cases who tested QFT-Plus negative were known to be immunocompromised. Three contacts with TB disease tested positive in the TB1 tube only, 13 tested positive in the TB2 tube only, and 102 tested positive in both TB1 and TB2 tubes. The TB2 tube increased the sensitivity of the assay from 84.6% to 95.2% for TB disease. Compared to the 2,800 contacts with TBI who tested positive on their first QFT-Plus or who were non-stringent converters (“All Others with TBI”), a significantly higher proportion of contacts with TB disease tested positive in the TB2 tube (97.5% vs 92.7%, *P* = 0.049) (Table 2). Contacts with TB disease had significantly higher median IFN-γ levels in both TB1 and TB2 tubes compared to All Others with TBI (1.50 IU/mL vs 0.96 IU/mL, *P* = 0.003, and 1.88 IU/mL vs 1.06 IU/mL, *P* < 0.0001, respectively) (Table 2).

**TABLE 2:** Qualitative and quantitative QFT-PLUS results of contacts with TB disease and All Others with TBI (i.e., contacts with TBI excluding Stringent Converters)^*c*^

Qualitative QFT-Plus result	TB disease *N* = 118^*a*^ (%)	All Others with TBI *N* = 2,800 (%)	Chi-squared *P*-value
TB1 and TB2 positive	102 (86.4%)	2,247 (80.3%)	0.096
TB1 positive	105 (89.0%)	2,451 (87.5%)	0.640
TB2 positive	115 (97.5%)	2,596 (92.7%)	0.049
TB2-TB1 >0.6 IU/mL^*b*^	34 (28.8%)	353 (12.6%)	<0.0001
Median QFT-Plus titer IU/mL (range)			
TB1-Nil	1.50 (−0.20 to 66.07)	0.96 (−0.52 to 153.30)	0.003
TB2-Nil	1.88 (−0.01 to 66.07)	1.06 (−0.82 to 153.30)	<0.0001
TB2-Nil-TB1-Nil	0.13 (−2.30 to 59.76)	0.06 (−62.02 to 62.44)	<0.0001

^*a*^ Excludes six contacts with active TB with negative QFT-Plus result.

^*b*^ Among contacts who were positive in either or both TB1 and TB2 tubes.

^*c*^ *P* < 0.05 is taken as statistically significant.

A significantly higher proportion of Stringent Converters tested positive in the TB2 tube than All Others with LTBI (97.5% vs 92.7%, *P* = 0.019 (Table 3). The median IFN-γ responses in both TB1 and TB2 tubes were significantly higher in Stringent Converters compared to All Others with TBI (1.24 IU/mL vs 0.96 IU/mL, *P* = 0.017 and 1.32 IU/mL vs 1.06 IU/mL, *P* < 0.0001 respectively) (Table 3).

**TABLE 3:** Qualitative and quantitative QFT-PLUS results of Stringent Converters and All Others with TBI (i.e., contacts with TBI excluding Stringent Converters)^*b*^

Qualitative QFT-Plus result	Stringent Converters *N* = 163	All Others with TBI *N* = 2,800	Chi-squared *P*-value
TB1 and TB2 positive	143 (87.7%)	2,247 (80.3%)	0.02
TB1 positive	147 (90.2%)	2,451 (87.5%)	0.317
TB2 positive	159 (97.5%)	2,596 (92.7%)	0.019
TB2-TB1 >0.6 IU/mL^*a*^	38 (23.3%)	353 (12.6%)	<0.0001
Median QFT-Plus titer IU/mL (range)			
TB1-Nil	1.24 (−0.13 to 55.07)	0.96 (−0.52 to 153.30)	0.017
TB2-Nil	1.32 (−0.25 to 55.07)	1.06 (−0.82 to 153.30)	<0.0001
TB2-Nil-TB1-Nil	0.17 (−10.27 to 5.00)	0.06 (−62.02 to 62.44)	0.036

^*a*^ Among contacts who were positive in either or both TB1 and TB2 tubes.

^*b*^ *P* < 0.05 is taken as statistically significant.

### CD8 T cell responses

A significantly higher proportion of contacts with TB disease had TB2-TB1 IFN-γ >0.6 IU/mL (28.8% vs 12.6%, *P* < 0.0001) compared to All Others with TBI. The median TB2-TB1 IFN-γ titer was also significantly higher in contacts with TB disease (0.13 vs 0.06 IU/mL, *P* < 0.0001) (Table 2). Contacts with TB disease were significantly more likely to have TB2-TB1 IFN-γ >0.6 IU/mL compared to all QFT-Plus-positive contacts without TB disease (OR 2.663, CI 1.763–4.021, *P* < 0.001) (Table 4).

**TABLE 4:** Factors associated with CD8 response (TB2-TB1 IFN-γ >0.6 IU/mL) in contacts with TBI^*a*^^,*b*^

	Number/proportion with TB2-TB1 IFN-γ >0.6 IU/mL	Univariable model	
		cOR (95% CI)	*P*-value
Stringent QFT-Plus Converters (*n* = 163) All others with TBI (*n* = 2,800)	38 (23.3%) 353 (12.6%)	2.11 (1.44–3.08) Reference	<0.001
Age <60 years (*n* = 2,235) Age ≥60 years (*n* = 728)	301 (13.5%) 90 (12.4%)	0.91 (0.71–1.17) Reference	0.444
Foreign-born (*n* = 1,361) Local-born (*n* = 1,602)	174 (12.8%) 217 (13.5%)	1.07 (0.86–1.32) Reference	0.542
Diabetes (*n* = 283) No diabetes (*n* = 2,680)	36 (12.7%) 355 (13.2%)	0.96 (0.66–1.38) Reference	0.804

^*a*^ cOR, crude odds ratio; aOR, adjusted odds ratio.

^*b*^ *P* < 0.05 is taken as statistically significant.

Compared to All Others with TBI, a significantly higher proportion of Stringent Converters tested positive in the TB2 tube (97.5% vs 92.7%, *P* = 0.019) and had TB2-TB1 IFN-γ >0.6 IU/mL (23.3% vs 12.6%, p<0.0001) (Table 3). Stringent Converters were significantly more likely to have TB2-TB1 IFN-γ >0.6 IU/mL compared to All Others with TBI (OR 2.107, CI 1.441–3.081, *P* < 0.001) (Table 4).

Stringent conversion and TB disease were associated with CD8+ response (crude OR [cOR] 2.11, 95% CI 1.44–3.08, *P* < 0.001 and cOR 2.66, 95% CI 1.76–4.02, *P* < 0.001, respectively). Age, country of birth (local-born vs foreign-born), and diabetes were not associated with CD8+ IFN-γ responses (Table 4). Multivariate analyses showed that stringent conversion and TB disease were independently associated with CD8+ response (adjusted OR [aOR] 2.09, 95% CI 1.43–3.06, *P* < 0.001 and aOR 2.67, 95% CI 1.76–4.02, *P* < 0.001 respectively).

## DISCUSSION

Our study in a population of 19,397 contacts screened with the QFT-Plus at the Singapore TB Contact Clinic showed that those with TB disease and Stringent QFT-Plus Converters (i.e., those highly likely to have recent infection) were significantly more likely to demonstrate CD8+ responses as indicated by TB2-TB1 IFN-γ >0.6 IU/mL, as compared to All Others with TBI. This is in keeping with *in vitro* findings that CD8+ T cells preferentially recognize heavily infected macrophages, which would be expected to predominate in TB disease and in recent infection, as opposed to remote TBI in which *M. tb* infection is effectively contained by the host immune system. Our findings suggest the possible clinical utility of the QFT-Plus assay CD8+ response to indicate *M. tb* activity in hosts who may not have clear-cut signs and symptoms, to provide guidance on whether to investigate and/or to treat as for TB disease. An increased QFT-Plus CD8+ response may also be useful clinically to distinguish persons with a high likelihood of recent TBI (an important risk factor for progression to TB disease), who are candidates for TPT, from those with TBI acquired in the remote past, in whom the risk of progression is markedly diminished.

Our findings pertaining to CD8+ responses in TB disease are consistent with that of a meta-analysis by Darmawan et al., which showed the mean difference of IFN-γ production between TB2 and TB1 tubes to be significantly higher in TB disease than in TBI (15). This meta-analysis, however, found IFN-γ production to be lower in both TB1 and TB2 tubes in active TB subjects than in those with TBI.

Early reports in small numbers of subjects suggested a correlation between CD8+ T cell response and recent TBI (16, 17). Subsequent studies utilizing TB2-TB1 IFN-γ >0.6 IU/mL as a marker of CD8+ T cell response and using proximity to the index case, exposure hours, country of origin as surrogates for recent infection reported mixed findings. Machado et al. in Portugal showed CD8+ T cell activity to be higher among persons recently exposed to confirmed index cases, with an association seen with positive sputum smear of the index case and exposure time (18). This study did not include QFT-Plus converters. A study in Moldova by Pan et al. showed that strong CD8+ responses correlated with index cases’ infectiousness, and that a TB1 IFN-γ ≥0.03 IU/mL combined with a TB2 IFN-γ ≥0.06 IU/mL was predictive of a 19-fold increase for QFT-Plus stringent conversion (19). In contrast, a European multicenter study found that the IFN-γ responses and differences in the TB1 and TB2 tubes were not significantly different between a group at high risk of recent exposure (368 contacts), a group without known risk of recent exposure (229 patients with immune-mediated inflammatory diseases), and another group with indeterminate risk of recent exposure (89 asylum seekers or persons from abroad) (20). This study included 11 contacts with QFT-Plus conversion, among whom only one (6.7%) demonstrated TB2-TB1 IFN-γ >0.6 IU/mL in the second test. The prognostic value of the QFT-Plus in predicting incident TB was prospectively studied among contacts in London. The authors found that the difference between TB1 and TB2 responses did not predict incident TB (21). A more recent study on adolescents and young adults in a high TB burden setting (Zambia and South Africa) compared 274 QFT-Plus converters (recent infection) with 1,069 who tested baseline and repeat positive (remote infection) (22). This study found higher mean differential TB2-TB1 responses in those with recent versus remote TBI, but this did not reach statistical significance (0.01 IU/mL vs −0.22 IU/mL, *P* = 0.145). Interestingly, and in contrast to our findings, this study showed higher TB1 and TB2 responses in remote than recent infection, although greater TB2 responses than TB1 responses in those with recent infection were found. An explanation provided by the authors for the higher TB1 and TB2 IFN-γ responses in their remote infection group was the possibility of re-infection during the follow-up period in the extremely high TB prevalence setting of communities in South Africa.

The strength of our study was the large cohort screened under a national program, enabling the analysis of CD8+ T cell responses in a relatively large number of stringent QFT-Plus converters. There were several study limitations. As our study population did not include immunocompromised persons (who were screened with the T-SPOT.*TB* under the national TB program), our data may be biased toward stronger IFN-γ responses. The higher quantitative IFN-γ and TB2-TB1 IFN-γ titers in contacts with TB disease and recent TBI, although statistically significant, may also not be clinically useful in view of the small differences in IFN-γ values. Prospective studies need to be done to evaluate the utility of the assay in clinical settings. We did not have information on the contacts’ exposure hours to further stratify risk for recent TBI. We also did not have a control group of non-contacts who would have been clearly likely to have remote TBI for comparison. However, although our analysis was performed in a contact screening program, it is not unreasonable to assume that a fair proportion of contacts who tested positive on their first QFT-Plus test may have been infected from previous exposure (i.e., have remote TBI) in view of our country’s intermediate incidence setting (23).

In conclusion, our study showed that the QFT-Plus assay CD8+ T cell response was significantly associated with TB disease and recent TBI. This adds to the evidence for the potential utility of this marker to identify persons who would benefit from further investigations for TB disease or who would be candidates for TPT.

## References

[1] Houben RMGJ, Dodd PJ. 2016. The global burden of latent tuberculosis infection: a re-estimation using mathematical modelling. PLoS Med 13:e1002152. doi:10.1371/journal.pmed.1002152 27780211 PMC5079585

[2] Uplekar M, Weil D. 2015. Lonnroth K et al WHO’s new End TB strategy. Lancet 285:1799–1801. 10.1016/S0140-6736(15)60570-0 25814376

[3] Alonzi T, Petruccioli E, Aiello A, Repele F, Goletti D. 2025. Diagnostic tests for tuberculosis infection and predictive indicators of disease progression: utilizing host and pathogen biomarkers to enhance the tuberculosis elimination strategies. Int J Infect Dis 155:107880. doi:10.1016/j.ijid.2025.107880 40086617

[4] Lewinsohn DA, Heinzel AS, Gardner JM, Zhu L, Alderson MR, Lewinsohn DM. 2003. Mycobacterium tuberculosis-specific CD8+ T cells preferentially recognize heavily infected cells. Am J Respir Crit Care Med 168:1346–1352. doi:10.1164/rccm.200306-837OC 12969871

[5] Rozot V, Vigano S, Mazza-Stalder J, Idrizi E, Day CL, Perreau M, Lazor-Blanchet C, Petruccioli E, Hanekom W, Goletti D, Bart P-A, Nicod L, Pantaleo G, Harari A. 2013. Mycobacterium tuberculosis-specific CD8+ T cells are functionally and phenotypically different between latent infection and active disease. Eur J Immunol 43:1568–1577. doi:10.1002/eji.201243262 23456989 PMC6535091

[6] Petruccioli E, Chiacchio T, Peppono I, et al.. 2016. Firsts characterization of the CD4 and CD8 T-cell responses to QuantiFERON-TB plus. J Infect 73:588–597. doi:10.1016/j.inf.2016.09.008 27717779

[7] Qiagen. 2023. QuantiFERON-TB Gold plus (QFT-Plus) ELISA Package Insert. Qiagen, Germantown, MD.

[8] Shafeque A, Bigio J, Hogan CA, Pai M, Banaei N. 2020. Fourth-generation QuantiFERON-TB gold plus: What is the evidence? J Clin Microbiol 58:10. doi:10.1128/JCM.01950-19 PMC7448650 32493779

[9] Oh CE, Ortiz-Brizuela E, Bastos ML, Menzies D. 2021. Comparing the diagnostic performance of QuantiFERON-TB gold plus to other tests of latent tuberculosis infection: a systematic review and meta-analysis. Clin Infect Dis 73:e1116–e1125. doi:10.1093/cid/ciaa1822 33289038 PMC8423471

[10] Metcalfe JZ, Cattamanchi A, McCulloch CE, Lew JD, Ha NP, Graviss EA. 2013. Test variability of the QuantiFERON-TB gold in-tube assay in clinical practice. Am J Respir Crit Care Med 187:206–211. doi:10.1164/rccm.201203-0430OC 23103734 PMC3570654

[11] Chee CBE, Teleman MD, Boudville IC, Do SE, Wang YT. 2004. Treatment of latent TB infection for close contacts as a complementary TB control strategy in Singapore. Int J Tuberc Lung Dis 8:226–231. 15139452

[12] Chee CBE, Emmanuel SC, Wang YT. 1997. A brave STEP forward – the Singapore Tuberculosis elimination programme. Singapore Med J Vol 38:359–361. 9407757

[13] Chee CBE, James L. 2003. The Singapore Tuberculosis Elimination Programme: the first five years. Bull World Health Organ 81:217–221. 12764518 PMC2572427

[14] Nemes E, Rozot V, Geldenhuys H, Bilek N, Mabwe S, Abrahams D, Makhethe L, Erasmus M, Keyser A, Toefy A, Cloete Y, Ratangee F, Blauenfeldt T, Ruhwald M, Walzl G, Smith B, Loxton AG, Hanekom WA, Andrews JR, Lempicki MD, Ellis R, Ginsberg AM, Hatherill M, Scriba TJ, C-040-404 Study Team and the Adolescent Cohort Study Team. 2017. Optimization and interpretation of serial QuantiFERON testing to measure acquisition of Mycobacterium tuberculosis infection. Am J Respir Crit Care Med 196:638–648. doi:10.1164/rccm.201704-0817OC 28737960 PMC5620669

[15] Darmawan G, Liman LMS, Hamijoyo L, Atik N, Alisjahbana B, Sahiratmadja E. 2023. Comparison of interferon-gamma production between TB1 and TB2 tubes of QuantiFERON-TB Gold Plus: a meta-analysis. Clinical Chemistry and Laboratory Medicine (CCLM) 61:2067–2075. doi:10.1515/cclm-2023-0293 37221870

[16] Nikolova M, Markova R, Drenska R, Muhtarova M, Todorova Y, Dimitrov V, Taskov H, Saltini C, Amicosante M. 2013. Antigen-specific CD4- and CD8-positive signatures in different phases of Mycobacterium tuberculosis infection. Diagn Microbiol Infect Dis 75:277–281. doi:10.1016/j.diagmicrobio.2012.11.023 23276770

[17] Barcellini L, Borroni E, Brown J, Brunetti E, Campisi D, Castellotti PF, Codecasa LR, Cugnata F, Di Serio C, Ferrarese M, Goletti D, Lipman M, Rancoita PMV, Russo G, Tadolini M, Vanino E, Cirillo DM. 2016. First evaluation of QuantiFERON-TB GOLD Plus performance in contact screening. Eur Respir J 48:1411–1419. doi:10.1183/13993003.00510-2016 27390280

[18] Viana Machado F, Morais C, Santos S, Reis R. 2021. Evaluation of CD8+ response in QuantiFERON-TB Gold Plus as a marker of recent infection. Respir Med 185:106508. doi:10.1016/j.rmed.2021.106508 34171790

[19] Pan S-W, Catanzaro DG, Seifert M, Syed RR, Hillery N, Ho M-L, Crudu V, Tudor E, Ciobanu N, Codreanu A, Catanzaro A, Rodwell TC. 2023. Predicting stringent QuantiFERON-TB Gold Plus conversions in contacts of tuberculosis patients. J Microbiol Immunol Infect 56:1073–1083. doi:10.1016/j.jmii.2023.07.014 37580184 PMC10604336

[20] Perez-Recio S, Pallares N, Grijota-Camino MD, et al.. 2021. Identification of recent tuberculosis exposure using QuantiFERON-TB Gold Plus, a multicentre study. MicrobiolSpectrum:e00972–21. 10.1128/Spectrum.00972-21 PMC8579846 34756079

[21] Gupta RK, Kunst H, Lipman M, Noursadeghi M, Jackson C, Southern J, Imran A, Lozewicz S, Abubakar I. 2020. Evaluation of QuantiFERON-TB Gold Plus for predicting incident tuberculosis among recent contacts: a prospective cohort study. Ann Am Thorac Soc 17:646–650. doi:10.1513/AnnalsATS.201905-407RL 32083944 PMC7193805

[22] Amofa-Sekyi M, Schaap A, Mureithi L, Kosloff B, Cheeba M, Kangololo B, Vermaak R, Paulsen R, Ruperez M, Floyd S, de Haas P, Fidler S, Hayes R, Ayles H, Shanaube K, TREATS study team. 2024. Comparing patterns of recent and remote Mycobacterium tuberculosis infection determined using the QuantiFERON-TB Gold Plus assay in a high TB burden setting. PLoS Glob Public Health 4:e0003182. doi:10.1371/journal.pgph.0003182 38768253 PMC11104639

[23] Yap P, Tan KHX, Lim WY, Barkham T, Tan LWL, Chen MI-C, Wang YT, Chee CBE. 2018. Prevalence of and risk factors associated with latent tuberculosis in Singapore: a cross-sectional survey. Int J Infect Dis 72:55–62. doi:10.1016/j.ijid.2018.05.004 29758278

