# Erratum for Chee et al., “QuantiFERON-TB Gold Plus CD8+ T cell responses in contacts with tuberculosis disease and recent tuberculosis infection”

**DOI:** 10.1128/spectrum.01512-26

**Published:** 2026-08-25

**Authors:** Cynthia Bin-Eng Chee, KhinMar Kyi-Win, Shera Tan, Yee-Tang Wang

## ERRATUM

Volume 13, no. 12, e01353-25, 2025, https://doi.org/10.1128/spectrum.01353-25. Abstract, sentence 4: “...from January 2018 to October 2018” should read “...from January 2017 to October 2018.”

Introduction, last paragraph, sentence 3: “...the QFT-Plus from December 2017” should read “...the QFT-Plus from December 2016.”

Introduction, last paragraph, sentence 4: “...from January 2018 to October 2018” should read “...from January 2017 to October 2018.”

Figure 1, top box: “...1 Jan 2018 to 31 Oct 2018” should read “...1 Jan 2017 to 31 Oct 2018.”

